# Inhibition of Influenza A Virus (H1N1) Fusion by Benzenesulfonamide Derivatives Targeting Viral Hemagglutinin

**DOI:** 10.1371/journal.pone.0029120

**Published:** 2011-12-15

**Authors:** Lei Zhu, Yuhuan Li, Shaohua Li, Haodong Li, Zongxing Qiu, Chichang Lee, Henry Lu, Xianfeng Lin, Rong Zhao, Li Chen, Jim Z. Wu, Guozhi Tang, Wengang Yang

**Affiliations:** 1 Roche Pharma Research and Early Development, Shanghai, China; 2 WuXi AppTec Co., Ltd., Shanghai, China; 3 Institute of Medicinal Biotechnology, Chinese Academy of Medical Sciences, Beijing, China; University of Hong Kong, Hong Kong

## Abstract

Hemagglutinin (HA) of the influenza virus plays a crucial role in the early stage of the viral life cycle by binding to sialic acid on the surface of host epithelial cells and mediating fusion between virus envelope and endosome membrane for the release of viral genomes into the cytoplasm. To initiate virus fusion, endosome pH is lowered by acidification causing an irreversible conformational change of HA, which in turn results in a fusogenic HA. In this study, we describe characterization of an HA inhibitor of influenza H1N1 viruses, RO5464466. One-cycle time course study in MDCK cells showed that this compound acted at an early step of influenza virus replication. Results from HA-mediated hemolysis of chicken red blood cells and trypsin sensitivity assay of isolated HA clearly showed that RO5464466 targeted HA. In cell-based assays involving multiple rounds of virus infection and replication, RO5464466 inhibited an established influenza infection. The overall production of progeny viruses, as a result of the compound's inhibitory effect on fusion, was dramatically reduced by 8 log units when compared with a negative control. Furthermore, RO5487624, a close analogue of RO5464466, with pharmacokinetic properties suitable for *in vivo* efficacy studies displayed a protective effect on mice that were lethally challenged with influenza H1N1 virus. These results might benefit further characterization and development of novel anti-influenza agents by targeting viral hemagglutinin.

## Introduction

Influenza A viruses are enveloped RNA viruses. Their genomes are composed of eight single-stranded, negative-sensed RNA segments. These viruses cause respiratory diseases in humans and animals with a high morbidity and mortality [1]. The influenza pandemic of 1918, also known as the Spanish flu, is believed to have killed 20 million humans [2]. The reassortment of avian flu RNA fragments with circulating human viruses caused the other two pandemics, the 1957 H2N2 “Asian influenza” and the 1968 H3N2 “Hong Kong influenza” [3], [4]. Human beings are facing the challenges of influenza from various directions. Seasonal influenza epidemics affect about 5–15% of the world's population. Its complications result in an annual mortality ranging from 250,000 to 500,000. Infection of avian flu strains, mostly H5N1, has been reported in many Asian countries. Though no frequent human-to-human spreading has been documented, avian flu infection was serious and associated with a high mortality of ∼60% of infected persons [5], [6]. In early April 2009 a new swine-origin influenza virus (S-OIV), A (H1N1), emerged in Mexico. The virus quickly spread worldwide through human-to-human transmission. In June 2009, the World Health Organization raised the influenza pandemic alert to the highest level (level 6) [7].

Currently, seasonal trivalent influenza vaccines and vaccines specific for H5N1 and swine flu are either available or in clinical trials. Prophylaxis is an effective method, at least in some populations, for preventing influenza virus infection and its potentially severe complications. However, due to continuous viral antigenicity shifting and drifting that make prediction of future circulating flu strain antigens difficult, and due to the challenges of rapid mass vaccine production of vaccines during a pandemic, other anti-influenza therapeutics including small molecule drugs are highly desirable [8]. There are currently two types of anti-influenza medicines on the market, influenza neuraminidase inhibitors, oseltamivir phosphate (Tamiflu) and zanamivir (Relenza) and viral M2 ion channel blockers amantadine and rimantadine [1]. During 2009 pandemic H1N1 flu, an experimental neuraminidase inhibitor, peramivir, had been issued by FDA for an emergency use for hospitalized patients only in cases where other treatments are ineffective or unavailable [9]. This authorization expired on June 23, 2010. An apparent limitation of currently available antivirals is the risk of development of drug resistance that has been frequently reported for both neuraminidase and M2 channel inhibitors. To overcome/attenuate the appearance of drug-resistant viruses and increase the effectiveness of current anti-flu drugs, it is urgent to discover therapeutics with a new mechanism of anti-influenza action that can be used as therapeutic or prophylactic agents either alone or combined with current antiviral drugs.

Hemagglutinin (HA) is a glycoprotein located on the envelope of influenza virus particles [10]. Currently, 16 hemagglutinin subtypes of influenza A viruses have been reported that fall into two major phylogenetic groupings: group 1 (H1, H2, H5, H6, H8, H9, H11, H12, H13, and H16) and group 2 (H3, H4, H7, H10, H14, and H15). HA_0_, the precursor of HA, is synthesized as a single polypeptide and cleaved at a specific site by a cellular protease to yield two subunits, HA_1_ and HA_2_. Individual HA_1_ and HA_2_ are linked by a disulfide bond. Three HA_1_-HA_2_ dimers form a homotrimer that is a functional unit of hemagglutinin [11]–[13]. The life cycle of influenza virus infection begins with the binding between the receptor binding pocket located in the membrane distal region of HA and sialic acid on the surface of host epithelial cells. Depending on the type of infected host species, one of the two types of sialic acids, *N*-acetylneuraminic acid α (2,3)-Gal or *N*-acetylneuraminic acid α (2,6)-Gal, is preferably recognized by HA protein. After HA-receptor binding, influenza virus enters into the cell by endocytosis resulting in the formation of a virus-containing endosome. To release virus genomes into the cytoplasm, fusion between viral envelope and endosome membrane occurs in an acidic environment that triggers an irreversibly conformational change of HA in which the hydrophobic fusion peptide at the N-terminus of HA_2_ is released from a buried position. The exposed HA_2_ fusogenic domain interacts with endosomal membrane, initiates a series of structural rearrangements of HA_2_, and finally leads to membrane fusion and release of viral RNA genomes [10], [14]–[16]. The viral genomes of RNA-protein complexes (RNP) are further translocated into the nucleus where viral RNA replication occurs.

Interfering HA-mediated fusion has become a strategy of anti-influenza drug discovery for many years. To date, there are two types of fusion inhibitors that block influenza infection by either nonspecifically increasing endosome pH or directly targeting HA protein. Examples of the first mechanism include chloroquine [17] and triperiden [18]. By interfering with H^+^ pumping on the endosome membrane, these molecules elevate the energy barrier for endosome acidification. When intra-endosomal pH fails to approach the required critical point HA conformational change does not happen and virus fusion is abolished. In the second mechanism of fusion inhibition, a small molecule inhibitor directly binds to HA and blocks HA conformational change. Examples of fusion inhibitors in this second category are BMY 27709 [19]–[21], 180299 (a podocarpic acid derivative, [22]), *tert*-butyl hydroquinone (TBHQ) [23], [24], N-substituted piperidine [25], and stachyflin [26]. These inhibitors are predominantly influenza virus group-specific, meaning they are active against either group 1 or group 2, but not both, influenza viruses. Except for TBHQ, the binding sites of these inhibitors on HA are unknown, although drug resistance profiling and photoaffinity labeling studies suggest that these molecules bind to HA_2_ protein. So far, no preclinical study of these fusion inhibitors including *in vivo* efficacy in an animal model was reported. One exception of the second class of influenza fusion inhibitors is arbidol, which exhibits a broad spectrum of anti-viral activities and has been proposed to target HA based on mechanism of action and drug resistance studies. This compound has been used in the clinic to treat influenza infection in Russia [27], [28] .

Recently, the binding pocket of *tert*-butyl hydroquinone (TBHQ) on HA_2_ peptide of both H3 and H14 strains has been determined in a co-crystallization study [24]. It is a hydrophobic pocket at the interface between HA_2_ monomers in a prefusion HA trimer. By blocking HA fusogenic conformational changes in the low pH environment of the endosome, TBHQ inhibits fusion of group 2 influenza viruses.

In this study, characterization of an HA inhibitor of influenza H1N1 viruses, RO5464466, is described. Based on the results of time course studies in one replication cycle and mechanism of action studies, this compound is believed to directly target HA and block fusion by stabilizing the pre-fusion structure of hemagglutinin. In cell-based assays involving multiple rounds of virus infection and replication, RO5464466 inhibited an established influenza infection. Moreover, RO5487624, an analogue of RO5464466, with pharmacokinetic properties for *in vivo* efficacy studies showed a protective effect on mice that were lethally challenged with influenza H1N1 virus. All of these data make it attractive to further characterize and develop HA inhibitors, including compounds shown in this report, for potential clinical use.

## Materials and Methods

### Ethics statement

All animal studies were approved by the Institutional Animal Care and Use Committee (IACUC) of Medicilon/MPI Research LLC with the approve ID of 18003-11021 or approved by the Ethics Committee for Animal Experiments of the Institute of Medicinal Biotechnology, Chinese Academy of Medical Sciences. Animal study was conducted in accordance with the current facility's Standard Operating Procedures (SOPs) and in compliance with the Animal Welfare Act. Researchers of Roche Pharma Research and Early Development monitored all activities in animal study including the IACUC approval, performance, and compliance.

### Compound synthesis

**Figure 1:**
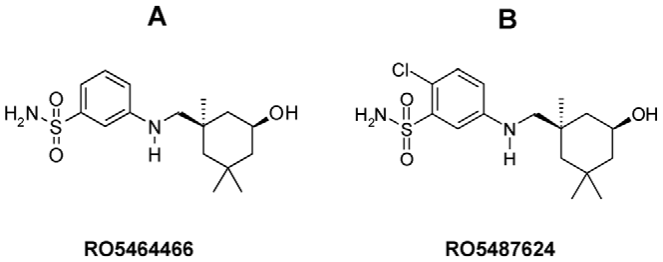
Chemical structure of RO5464466 (A) and RO5487624 (B), benzenesulfonamide derivatives. RO5464466 and RO5487624 were shown in [29] as compound 28 and 40, respectively.

RO5464466 {3-[(5-Hydroxy-1,3,3-trimethyl-cyclohexylmethyl)-amino]-benzenesulfonamide} and its derivative compound RO5487624 were synthesized at Roche Pharma Research and Early Development, China (Fig. 1). The carboxylic acid form of Oseltamivir (Tamiflu) was provided by Roche global inventory as phosphate salt. Oseltamivir was also provided by Shanghai Institute of Materia Medica, Chinese Academy of Sciences. Ribavirin was ordered from TCI (Shanghai) Development Co., Ltd, China.

### Cells and viruses

Madin-Darby canine kidney (MDCK) cells were purchased from American type culture collection (ATCC) and were maintained in minimal essential medium (MEM) containing 10% fetal bovine serum and antibiotics. Influenza A/Weiss/43 (H1N1), A/PR/8/34 (H1N1), A/Mal/302/54 (H1N1), A/New Jersey/8/76 (H1N1), and A/Hongkong/8/68 (H3N2) were purchased from ATCC. Influenza A/Human/Hubei/3/2005 was provided by the Institute of Virology of Wuhan, China. Influenza A/FM/1/47 mouse adapted strain was provided by the Institute of Virology, Chinese Academy of Preventive Medicine. All virus strains used in this study were propagated in 10-day-old embryonated chicken eggs at 37°C. Virus was harvested 48 h after inoculation as pooled allantoic fluid. After a brief centrifugation (3,000 rpm at room temperature for 20 minutes) and virus titer measurement by a hemagglutination test, virus was aliquoted and stored at a −80°C freezer.

### Viral cytopathic effect (CPE) assay

To measure anti-influenza activity of compounds, MDCK cells in Gibco, OptiPRO™ SFM (Invitrogen, cat no: 12309) were seeded into 96-well plates at a density of 5,000 cells per well. Next day, compounds were dissolved and serially half-log diluted in dimethyl sulfoxide (*DMSO*) first, then further diluted (100 folds) with Gibco SFM containing trypsin. Compound preparations and virus (50 plaque formation units, pfu) were added into corresponding wells to make a multiplicity of infection (m.o.i.) at 0.01 and a final trypsin concentration of 2.5 µg/ml. Final compound concentrations were ranged from 0.3 µM to 100 µM in general or properly reduced if a compound's EC_50_ was below than 0.3 µM in an initial test. The testing plates also contained medium control, cell control, virus control, and compound toxicity control. After a 3-day treatment, cell viability was measured with MTT ((3-(4,5-dimethylthiazol-2-yl)-2,5-diphenyltetrazolium bromide). Briefly, 20 µl of MTT (5 mg/ml, diluted in culture medium) was added into each well and incubated at 37°C for 4 hours. Reduced MTT (formazan) was extracted with acidic isopropanol. Absorbance at wavelengths of 570 nm and 630 nm (OD_570_ and OD_630_) was read on a microtiter plate reader. After subtraction of background OD values, dose response curves of half-log concentration vs. percentage of protection were generated, from which half maximal effective concentration (EC_50_) and half maximal toxic concentration (CC_50_) were calculated.

### Plaque reduction assay

MDCK cell suspension in UltraMDCK serum free medium (Lonza, cat. no:12-749Q) was seeded into 6-well plates with a density of 6×10^5^ cells per well. When monolayer was confluent, culture medium was removed. 100 pfu of virus as well as compound prepared in culture medium containing 2.5 µg/ml trypsin were added into wells. Plates were incubated in a CO_2_ incubator for 1 hour with several intermittent rockings. After virus absorption, inoculums were removed and 3 ml of 0.5% low-melting point agarose overlay prepared in culture medium containing 2.5 µg/ml trypsin and compound was added into wells. After incubating plates at 37°C for 3 days, the cell monolayer was fixed with 4% paraformaldehyde for 1 hour. Then, agarose overlay was removed and cell monolayer was stained with 0.5% (w/v) crystal violet prepared in 5% ethanol. Plaques were counted by visual examination.

### Hemagglutination inhibition assay

Hemagglutination inhibition (HI) assay was employed to evaluate the effect of a compound on virus adsorption to target cells. Compound in PBS was mixed with an equal volume of influenza virus that was serially diluted by two-fold in PBS. After 1 hour incubation at room temperature, 50 µl of compound-virus preparation was mixed with an equal volume of 1% chicken erythrocyte suspension in PBS in a V bottom 96-well plate. The mixture was incubated for 1 hour at room temperature before observing erythrocyte aggregation on the plate.

### Hemolysis inhibition assay

Fresh chicken erythrocytes were washed twice with PBS and re-suspended to make a 2% (v/v) suspension in PBS that was stored at 4°C until use. 100 µl of compound diluted in PBS was mixed with an equal volume of virus in a 96-deepwell plate. Allantoic fluid harvested from virus-inoculated embryonated chicken eggs was used as virus preparation in hemolysis assay. After incubating virus-compound mixture at room temperature for 30 minutes, 200 µl of 2% chicken erythrocytes pre-warmed at 37°C was added. The mixture was incubated at 37°C for another 30 minutes. To trigger hemolysis, 100 µl of sodium acetate (0.5 M, pH 5.2) was added and mixed well with erythrocyte suspension. The mixture was incubated at 37°C for 30 minutes for HA acidification and hemolysis. To separate non-lysed erythrocytes, plates were centrifuged at the end of incubation at 1,200 rpm for 6 minutes. 300 µl of supernatant was transferred to another flat bottom 96-well plate. OD_540_ was read on a microtiter plate reader. Percentage of protection was calculated as [1- (mean of OD540_compound_ − mean of OD540_PBS_)/(mean of OD540_DMSO_ − mean of OD540_PBS_)]×100%, where mean of OD540_compound_, mean of OD540_PBS_, and mean of OD540_DMSO_ are the absorbance of compound-, virus-containing samples; the absorbance of no virus control samples; the absorbance of DMSO-,virus-containing samples, respectively. IC_50_ is defined as the compound concentration that generates 50% of the maximal protection. Special attention should be paid when a compound is colorful or is hemolytic. Suitable controls should be set up to adjust absorption backgrounds since both of those features of a compound could result in a false negative outcome in hemolysis inhibition assay.

### HA purification and trypsin protection assay

HA was purified by a modification of the published procedure [21]. After a brief centrifugation to remove insoluble materials, virus in 300 ml of allantoic fluid was pelleted by an ultracentrifugation of 38,500 rpm at 4°C for 2 hours. The virus was suspended in PBS and subjected to a second ultracentrifugation of 25,000 rpm at 4°C for 3 hours through a discontinuous sucrose gradient composed of 60%, 45%, and 30% (w/v) of sucrose. Virus concentrated between 45% and 60% sucrose gradients was recovered by puncturing the tube with a needle and drawing the cloudy virus fraction. After diluting virus-containing sucrose with PBS, the virus was pelleted and resuspended in 1 ml of buffer (100 mM Tris HCl, pH7.4; 1 mM EDTA; 1 mM 2-β-mercaptoethanol; and 2 mg/ml of bromelain). Virus was digested with bromelain in the solution above at 37°C for 16 hours to release HA from virus particles. After a centrifugation of 10,000 rpm at room temperature for 20 minutes, the supernatant was applied to a Sephacryl-400 gel filtration column equilibrated with PBS. Elution was carried out at a flow rate of 0.5 ml/min. Fractions containing bromelain-cleaved HA (BHA) were concentrated in a Centriprep-30 concentrator to a volume of 2 ml and then were diluted 10-fold with PBS. For a further purification, BHA-containing solution was loaded onto an ion exchange column, Q Sepharose FF. After washing the column with 20 mM phosphate buffer (monosodium phosphate - disodium phosphate, pH8.0), BHA was eluted with 20 mM phosphate buffer; 0.3 M NaCl. BHA was identified by sodium dodecyl sulfate-polyacrylamide gel electrophoresis (SDS-PAGE), and concentrated by a Centriprep-30 apparatus.

To measure if a compound stabilizes BHA native structure in an acidic environment, sensitivity of BHA to trypsin digestion was determined in which only conformationally-changed BHA resulting from a low pH treatment is cleavable by trypsin. 4–6 µg of purified BHA was incubated with compound or various controls at 31°C for 15 minutes. The mixture was adjusted with 0.25 M citrate (pH 4.2) to a final pH of 5.0, and incubated for another 15 minutes at 31°C. pH of the reaction was then neutralized with 0.25 M Tris.HCl, pH 9.0 to the final pH of 7.5. 2 µg of trypsin was added to each reaction and digestion was carried out for 30 minutes at 37°C. Trypsin-mediated HA cleavage was checked on a 10% SDS-PAGE gel that was stained with Coomassie blue G-250.

### Pharmacokinetic analysis

RO5487624, an analogue of RO5464466, was used in *in vivo* pharmacokinetic study. Male CD-1 mice were administrated either intravenously (i.v.) via the tail vein with 6% solutol and 94% saline formulation of RO5487624 at 10 ml/kg body weight to provide a dose of 2 mg/kg, or orally (p.o.) by gavage with a 0.445% microcrystalline cellulose, 0.055% sodium carboxymethyl cellulose and 1.6% lactose in distilled water at 10 ml/kg body weight to provide three doses of 30, 100, 200 mg/kg. Blood samples were collected via jugular vein puncture or vena cava at 5 min, 15 min, 30 min, 1 h, 2 h, 4 h, 6 h, 8 h, and 24 h after i.v. dose administration and at 10 min, 30 min, 1 h, 2 h, 4 h, 6 h, 8 h, and 24 h after p.o. dose administration, respectively. Blood samples were centrifuged at 4°C at 2,700 rpm for 15 minutes to allow plasma separation. Lung samples in orally dosing groups were collected at 1 h, 4 h, 8 h, and 24 h post administration. Samples were analyzed individually for RO5487624 by liquid chromatography-tandem mass spectrometry with a limit of quantitation of 1 ng/ml. Mean +/− standard deviation (n = 4) concentrations of RO5487624 were calculated per administration route, per dose level, and per sampling time. Plasma concentration versus time profiles were subjected to a noncompartmental pharmacokinetic analysis using validated WinNonlin software (Version 5.2, Pharsight Corporation, CA).

### *In vivo* efficacy study

Influenza A virus strain A/FM/1/47 (H1N1) was grown in embryonated eggs. LD_50_ (median lethal dose) was determined in mice after serial dilution of allantoic fluid. 40 LD_50_ were used for viral challenge in all groups by intranasal inoculation of Kunming female mice, 5–7 weeks old, anesthetized by isoflurane. RO5487624 (200 mg/kg and 50 mg/kg) were administrated orally, twice per day for 7 days. Oseltamivir (50 mg/kg) and ribavirin (50 mg/kg) were used as positive controls and administrated twice per day by either an oral or i.p route, respectively, for 7 days. The first dosage of all treatments was given either 1 h before (prophylactic) or 3 h after (post exposure prophylaxis) virus challenge. Survival of the infected animals (10 in each group) was monitored for duration of 14 days starting from virus challenge.

## Results

### Anti-influenza activity of RO5464466

RO5464466 is a representative benzenesulfonamide compound discovered at Roche Pharma Research and Early Development, China (Fig. 1).

*In vitro* anti-influenza activity of RO5464466 was measured and presented in Table 1. RO5464466 possessed similar activities against four H1N1 strains (A/Weiss/43, A/PR/8/34, A/Mal/302/54, and A/New Jersey/8/76). However, it failed to exhibit an apparent inhibitory effect on the replication of two H3N2 strains (A/Hongkong/8/68 and A/Human/Hubei/3/2005) at a concentration of 100 µM in a CPE assay. RO5487624 was synthesized when majority of studies of RO5464466 was done. RO5487624 showed a slightly better *in vitro* anti-influenza activity when compared to RO5464466 and better pharmacokinetic properties for *in vivo* efficacy study (described later). The detailed synthesis schemes and the structure-activity relationship (SAR) studies of benzenesulfonamides can be found in a recent publication [29].

**Table 1:** Comparison of *in vitro* anti-influenza activities of RO5464466 and RO5487624.

	CPE (EC_50_)^a^						CC_50_ b	Homelysis assay (IC_50_)	BHA trypsin sensitivity^c^
	A/New Jersey/8/76 (H1N1)	A/Mal/302/54 (H1N1)	A/Weiss/43 (H1N1)	A/PR/8/34 (H1N1)	A/Hongkong/8/68 (H3N2)	A/Human/Hubei/3/2005 (H3N2)			
RO5464466	0.28	0.66	0.21	0.58	>100	>100	>100	0.29	+
RO5487624	0.09	0.45	0.09	0.21	>100	>100	71	0.25	+

a : All numbers (EC_50_, CC_50_, and IC_50_) are means of three independent experiments and shown in micromolar concentrations;

Some data from A/Weiss/43 have been shown in [29].

b : MDCK cells were used;

c : + means nearly 100% protection of BHA from trypsin degradation in the presence of compounds (10 µM).

### RO5464466 inhibited an early step of influenza virus infection

**Figure 2:**
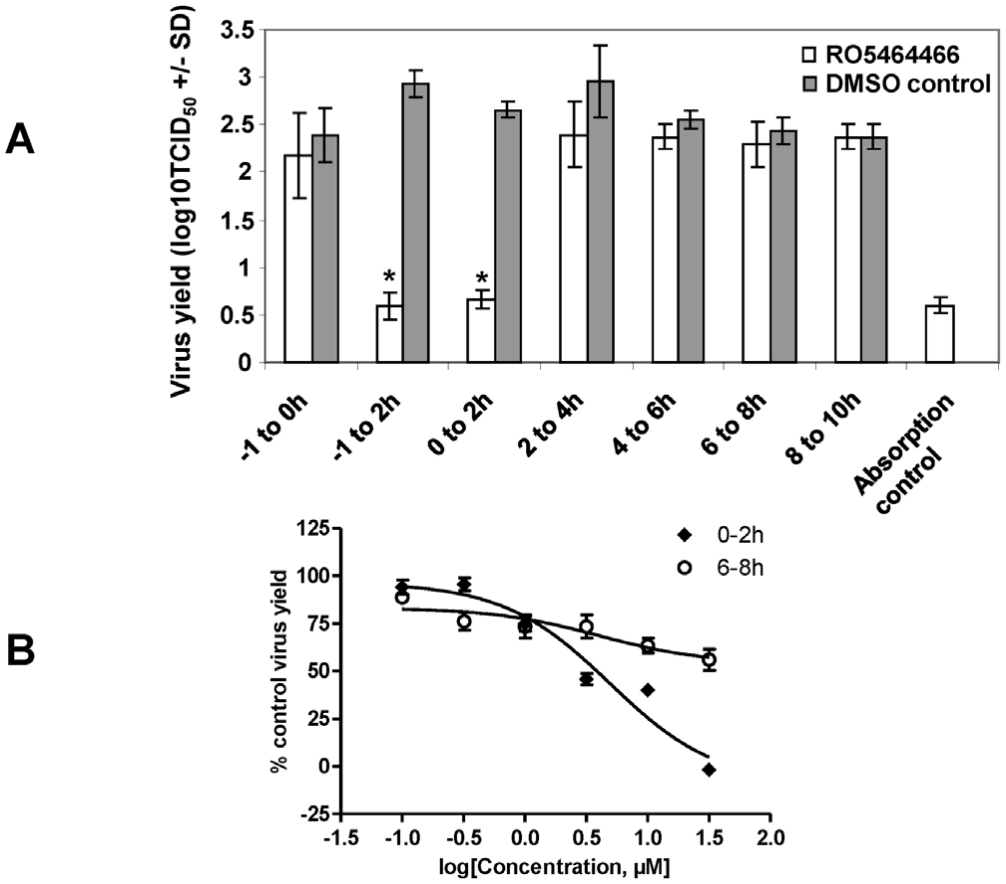
Time course of the inhibitory effect of RO5464466 in one cycle of influenza virus replication. (A) MDCK cells were seeded into 24-well plates at a density of 2×10^5^ cells per well. 140 pfu of influenza A/Weiss/43 (H1N1) were inoculated into each wells when the cell monolayer was confluent. After one hour of virus absorption on ice, culture wells were washed with PBS and the plates were incubated with medium containing 2 µg of trypsin per ml in a CO_2_ incubator at 37°C (0 hour post-infection). RO5464466 was added at −1 to 0 (absorption), −1 to 2, 0 to 2, 2 to 4, 4 to 6, 6 to 8, 8 to 10 hour post-infection at a final concentration of 20 µM. After each treatment period, the monolayer was washed and incubated in fresh medium until 10 hour post-infection. Every condition was tested in triplicate. Virus yield was determined by measuring TCID_50_ of the cell lysates. Open columns, the mean virus yield for cells treated with RO5464466; closed columns, the mean virus yield of DMSO controls; vertical lines, standard deviation (n = 3). One of three independent experiments was shown. *, results significantly different from that of each control by Student's t test (*P*<0.05). (B) Dose-response curves of RO5464466 at 0–2 and 6–8 hours post absorption intervals. Conditions of cell seeding and culturing, virus harvest, and TCID_50_ measurement were the same as described in (A). Virus-infected cell layer was treated with RO5464466 at concentrations ranging from 0.1 to 31.6 µM (six half-log diluted doses) for 2 hours at two intervals before incubated in no-compound fresh medium.

To identify the step(s) of influenza life cycle RO5464466 blocks, we performed a time course study of inhibitory effects of RO5464466. Since it takes 8 to 10 hours for influenza A/Weiss/43 virus to produce its progeny viruses after virus absorption, six 1 to 2-hour treatment intervals covering the whole life cycle of this virus strain from absorption to progeny production were used to disclose the time frame when RO5464466 exerts the maximal effect (Fig. 2A). The compound was applied during each of these intervals by adding compound and washing plates with PBS at specific time points. Following the treatment, fresh compound-free medium was added. DMSO (0.5% in culture medium) was used as a negative control and added/washed away at each time frame. All treated cells were harvested at a time point of 10 h post infection. Virus yield was determined by measuring TCID_50_ of every culture well in this experiment. As shown in Fig. 2A, apparent RO5464466-induced inhibition of virus amplification was observed when infected cells were treated in an interval of 0–2 hour post virus absorption. This time frame corresponds to early steps of influenza virus replication such as internalization or fusion. Virus yield measured in the 0–2 hour treatment group was similar to that of absorption control in which culture cells were placed into a −80°C freezer immediately after the virus absorption step to eliminate any possible virus replication and progeny production. These data suggest that RO5464466 completely blocks virus replication when administrated during the 0–2 hour post absorption interval. Any viruses that were detected in this group and absorption control group might derive from residual input viral particles that were not completely washed away after the absorption step. Treatment during other time frames did not significantly inhibit virus yield when compared to DMSO control groups. To confirm this result, we chose two time points, 0–2 hours and 6–8 hours post absorption intervals for a dose-response study. As shown in Fig. 2B, RO5464466 inhibited virus replication with an EC_50_ of 4 µM when added at 0–2 hours. This inhibitory effect was not apparent (EC_50_>31.6 µM) when the compound was added at 6–8 hours post absorption.

### Inhibitory effects of RO5464466 on HA-mediated hemolysis

**Figure 3:**
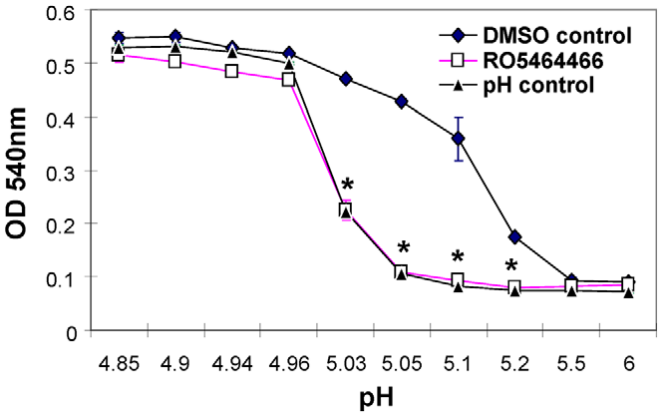
RO5464466 inhibited HA-mediated hemolysis of chicken erythrocytes. After mixing RO5464466 with virus-containing allantoic fluid, suspension of freshly prepared chicken erythrocytes was added. The mixture was then acidified with different pH from 4.85 to 6. The suspension was incubated at 37°C for 30 minutes. The final concentration of RO5464466 was 10 µM. After a brief spin, supernatants containing released hemoglobin were transferred to a second plate for measuring OD_540_. As a control, suspensions of erythrocytes alone were acidified (pH control) similarly to measure low pH-caused hemolysis. In the DMSO control group, every step was the same as described in RO5464466 group except the testing compound was omitted. All conditions were tested in duplicated wells. *, results significantly different (RO5464466 treated samples vs. DMSO controls) by Student's t test (*P*<0.05).

The early influenza virus replication steps include virus absorption, internalization of virus to form a virus-containing endosome, and fusion. Results in the time course study indicated that RO5464466 did not exert an inhibitory effect on virus absorption step (−1 to 0 hour interval in Fig. 2A). To further support this claim, hemagglutination-inhibition test was used in which aggregation of chicken erythrocytes was the consequence of the binding between viral hemagglutinin and sialic acid receptors on the surface of the cells. At a concentration of 10 µM, RO5464466 did not have an inhibitory effect on hemagglutination regardless of the viral dilutions used (data not shown), suggesting that RO5464466 doesn't interfere with virus-receptor interaction. To study if RO5464466 targets on virus fusion, a hemagglutinin-mediated hemolysis assay was employed, in which hemagglutinin of influenza virus binds to sialic acid receptors of chicken erythrocytes as described in the hemagglutination inhibition test. To trigger hemolysis, virus-cell suspension was acidified briefly to initiate HA conformational changes and generate “holes” on the surface of cells to release hemoglobin content from the cells. RO5464466, when added into virus-cell suspension before acidification, inhibited hemolysis in a dose-dependent manner with an IC_50_ of 0.29 µM (Table 1). To make sure that the inhibitory effect of RO5464466 on hemolysis could exist in a broad range of pH that was used to acidify virus–cell suspension, pH-dependence of hemolysis in the presence or absence of the compound was evaluated by adjusting virus-absorbed erythrocytes to different pH values before measuring the release of hemoglobin. As a control, erythrocyte suspension alone was also adjusted to the same pH to measure the extent of hemolysis caused by acidic pH itself. As shown in Fig. 3, when pH was lower than 5, hemolysis was triggered by acidic pH (pH control: no virus added to the suspension of chicken erythrocytes) to an extent where no difference was detected no matter if RO5464466 was present or not. On the other end, when pH was 5.5 or higher, no significant pH-mediated hemolysis was observed no matter if virus or compound exists, indicating HA conformational changes would not occur efficiently under these conditions. For the four pH values tested in the middle between 4.96 and 5.5, hemolysis caused by a low pH alone was nearly none or at a lower level when compared to the pH groups in the left end of Fig. 3. Under these pH values (5.03, 5.05, 5.1, and 5.2), adding influenza virus apparently increased the extent of hemolysis (shown as higher OD_540_ values in DMSO controls where virus and erythrocytes were present but there was no RO5464466). The overall hemolysis in DMSO controls might be composed of two different components: HA- mediated hemolysis under a low pH and hemolysis triggered by a low pH itself. In the presence of 10 µM RO5464466, HA-mediated hemolysis was completely blocked at all four pH values tested since the OD curve of RO5464466 was very close to the curve of pH control. This result indicates a possibility that RO5464466 may interfere with the acidification-induced conformational change of HA that is a prerequisite for virus-induced hemolysis. In addition, RO5464466 didn't result in a significant increase of OD_540_ when compared with the values observed in both pH and DMSO control groups at pH 5.5 and 6.0 (at the right end of Fig. 3) where no apparent hemolysis was observed. This data suggests RO5464466 itself is not hemolytic. As negative controls, two known influenza virus inhibitors, oseltamivir and ribavirin, did not show any effect on hemolysis (data not shown).

### RO5464466 protected hemagglutinin from trypsin digestion

**Figure 4:**
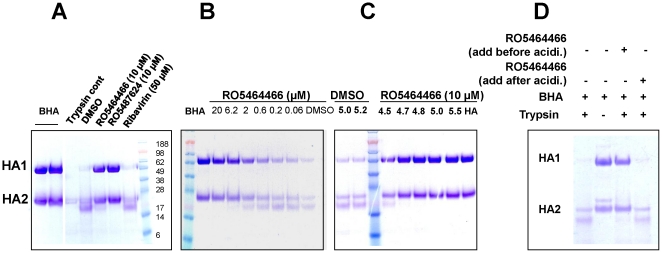
SDS-PAGE of trypsin sensitivity assay showing RO5464466 protected BHA from trypsin digestion in pH-dependent and dose-dependent manner. (A) 6 µg BHA was incubated with compounds of different concentrations for 15 minute at 31°C prior to acidification to pH 5.0 with 0.25 M citrate (pH 4.2). The mixture was neutralized to a final pH of 7.5 and treated with 2 µg of trypsin for 30 minutes at 37°C. The extent of trypsin cleavage on BHA was analyzed on a 10% SDS-PAGE gel. MW was shown on the right in thousands. Untreated BHA, trypsin alone, BHA trypsin digestion without a prior acidification step were used as controls and included in this figure. (B) Dose-dependent protection of BHA by RO5464466. (C) pH-dependent protection of BHA by RO5464466. After incubated with 10 µM of RO5464466, BHA was acidified to different final pH shown on the top of the gel before neutralization and trypsin digestion. As a negative control, DMSO-treated BHA was adjusted to two pH values (5.0 and 5.2) before neutralization and trypsin digestion. (D) RO5464466 protected BHA from trypsin digestion not by directly inhibiting trypsin enzymatic activity. RO5464466 was added into the reaction either before or after acidification of BHA.

To further confirm that the target of RO5464466 is HA, we studied hemagglutinin sensitivity to trypsin digestion in the presence or absence of RO5464466. Bromelain-cleaved HA (BHA) was isolated with a purity of ∼95% based on Coomassie blue staining of an SDS-PAGE gel (see Materials and Methods). It has been reported that a low pH (such as pH 5.2) environment triggers a characteristic conformational change of BHA, resulting in exposure of internal trypsin cleavage sites that renders BHA sensitive to trypsin digestion. As shown in Fig. 4A, BHA without acidification was resistant to trypsin digestion. However, after 15-minute incubation at pH5.2, both HA_1_ and HA_2_ in BHA were sensitive to trypsin. While ribavirin, a negative control, failed to show a protective effect on BHA, RO5464466 and RO5487624 completely protected HA from trypsin digestion. Fig. 4B shows that RO5464466 protected BHA in a dose-dependent manner with a half efficacious concentration of ∼1 µM.

To further study RO5464466's protective effect on BHA, pH of compound-BHA solution was adjusted from 5.5 to 4.5. After a brief incubation in acidic solution and a subsequent neutralization step, BHA was treated with trypsin as above. As shown in Fig. 4C, RO5464466 effectively protected BHA from trypsin-caused degradation throughout the tested pH values, except pH 4.5 where a slight trypsin-caused degradation of BHA was observed. This result reinforces the claim that RO5464466 stabilizes HA structure in a low pH environment.

To rule out the possibility that the protective effect of RO5464466 on trypsin-catalyzed degradation of BHA was due to direct inhibition of trypsin activity, RO5464466 was mixed with BHA either before or after acidification and neutralization steps, followed by trypsin digestion. As shown in Fig. 4D, the compound exerted a protective effect on BHA only when it was added into the reaction prior to acidification. Taken together, these results suggest that the compound binds to BHA and prevents its overall structure from low pH-triggered conformational changes.

### RO5464466 inhibited an established infection *in vitro*

**Figure 5:**
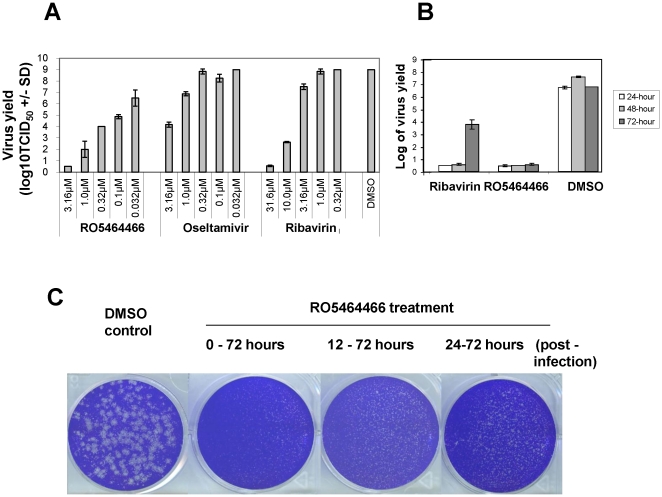
RO5464466 blocked the production of progeny virus and inhibited an established influenza virus infection. (A) Decrease of progeny virus production in cells treated with RO5464466 and other reference compounds. 12-well plates were seeded with MDCK cells at a density of 1.5×10^5^ cells per well. On the next day, serially diluted compounds and 150 pfu virus were added per well. 48 hours later, treatment was stopped and viral titer in cell monolayer was measured in TCID_50_. Virus yield was shown in a log scale, a mean of two wells. (B) Dynamics of virus yield reduction in the presence of inhibitors. In this experiment, all treatments began at the same time when virus was added into culture wells but were stopped at different time points of 24, 48, and 72 hours post-infection, respectively. (C) Inhibition of plaque formation by RO5464466. Details of seeding MDCK cells into 6-well plates and infecting monolayers with influenza A/Weiss/43 (H1N1) were described in the plaque reduction assay in the section of Materials and Methods. Instead of using 3 ml of agarose-containing overlay for each well, 2.5 ml of overlay was used. At each time point (0, 12, 24, 48 hours post-infection), 2.5 ml of culture medium that contained compound of 2-fold final concentration was added on the top of the overlay. At 72 hours post-infection, cell monolayers were fixed and stained to show plaques. Representative data of three independent experiments was shown.

To study anti-influenza virus activity of RO5464466 in more details, we used the production of progeny influenza virus as an end point to compare the *in vitro* activities of RO5464466 and other reference influenza reagents. After a treatment with RO5464466 at concentrations ranging from 0.0316 to 3.16 µM or oseltamivir at concentrations of 0.0316 to 3.16 µM or ribavirin at concentrations ranging from 0.316 to 31.6 µM for 48 hours, infected cells were subjected to a freeze-thaw to release viruses into the supernatant. All treatments including DMSO control were done in duplicate wells. TCID_50_ was determined to compare the yield of progeny virus produced from different treatment groups. As shown in Fig. 5A, RO5464466 treatment at 3.16 µM caused an 8-log reduction of virus yield. Ribavirin also strongly inhibited progeny virus production at concentrations of 10 and 31.6 µM. Oseltamivir at 3.16 µM resulted in a 5-log virus yield reduction. This effect was attenuated at the lower concentrations. In a second experiment, dynamics of progeny virus production in the presence of an inhibitor was studied. Infected MDCK cells were treated with RO5464466 and ribavirin at the highest concentration that was used in the previous experiment. At different time points of the treatment, virus was harvested and measured for the determination of progeny virus titer in each treatment group. In Fig. 5B, RO5464466 showed a strong inhibition at all time points of 24, 48, and 72 hours post infection. Progeny virus titer in the RO5464466 treated group was barely detectable at a baseline level. However, inhibitory effects of ribavirin became weaker as the treatment was elongated from 24 to 72 hours. These results suggest that RO5464466 has a stronger and longer-lasting *in vitro* anti-influenza activity than ribavirin.

In the experiments described above, RO5464466 and virus were added to the cell monolayer simultaneously. To mimic a real influenza infection situation in which virus infection occurs before pharmacological intervention, a plaque formation assay was designed to address if RO5464466 could inhibit overall virus amplification by blocking virus fusion in an established influenza infection, where the formation of a plaque is the result of multiple rounds of virus infection and replication initiated by a single infectious virus. In the following experiment, the timing of the addition of RO5464466 was different, ranging from immediate addition, 12 hours, 24 hours, or 48 hours after virus absorption. As shown in Fig. 5C, RO5464466 completely protected cell monolayer against cell death (no visible plaques) when it was added to cell cultures at both 0 and 12 hours post absorption. Addition of the compound at 24 hours post absorption still inhibited virus infection, reflected by the appearance of small plaques when compared with plaques observed in 48 hour post absorption or DMSO control groups. This result suggests that RO5464466, through inhibiting virus fusion, may significantly attenuate an established infection where multiple rounds of influenza virus infection and amplification were ongoing.

### Pharmacological properties and bioavailability of RO5487624

RO5487624 is a close analogue of RO5464466 (Fig. 1) and has *in vitro* anti-influenza activities similar to RO5464466. Its mechanism of action as an HA inhibitor was confirmed by trypsin sensitivity assay (Fig. 4A) and HA-mediated hemolysis assay (data not shown). We chose it for the following studies because RO5487624 possessed *in vivo* pharmacological properties better than that of RO5464466.

**Figure 6:**
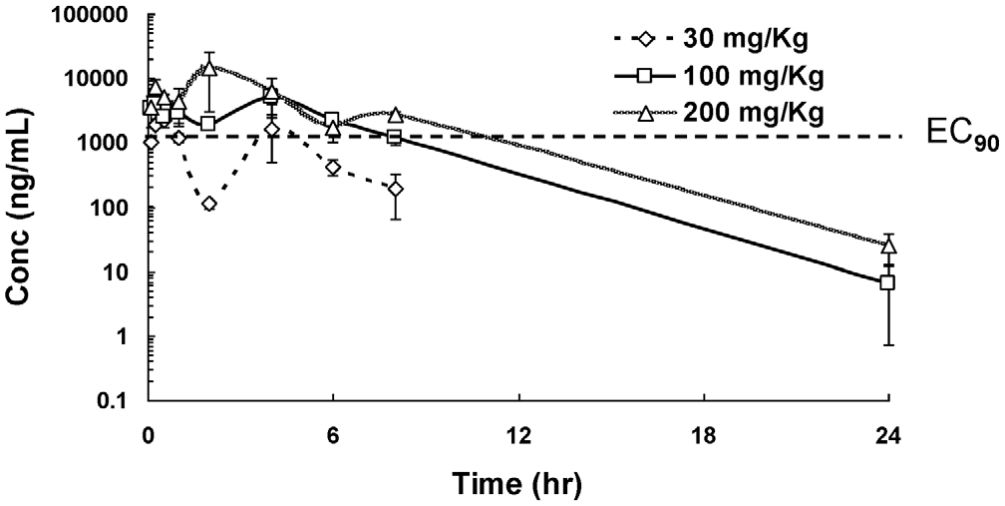
Pharmacokinetic analysis of RO5487624. Mean plasma concentration-time profiles of RO5487624 after an oral dose (30 mg/kg, ◊, 100 mg/kg □, and 200 mg/kg, Δ) to CD-1 mice. Vertical lines are standard deviation of each group (n = 3). Concentration corresponding to RO5487624's EC_90_ after protein binding adjustment is marked by a dotted line.

A single dose pharmacokinetics study of RO5487624 was performed in male CD-1 mice. After i.v. administration of RO5487624 at 2 mg/kg in Solutol-saline solution, plasma concentrations declined with a terminal half life (t1/2) of 2.5 hours, and total plasma clearance was 53 mL/min/kg, which is moderate. The mean values of AUC (0-t) and AUC (0-inf) were 587 and 629 ng*h/ml, respectively. In oral administrations of RO5487624 at 30, 100, 200 mg/kg, *C*_max_ and AUC were increased in a dose-dependent manner in plasma. Lung-plasma concentration ratios following oral administration were in a range of 1 to 3 in most time points and dosage groups. Based on the AUC (0-inf) values of intravenous and oral dose, the oral bioavailability of the compound in CD-1 mouse was estimated to be greater than 70%. After an adjustment of *in vitro* EC_90_ (0.3 µM) by compound's protein binding, plasma concentration of RO5487624 required to result in EC_90_ effect *in vivo* was calculated to be 3.75 µM. A corresponding line of this *in vivo* EC_90_ was shown in Fig. 6. It was estimated that at a single dose of 100 and 200 mg/kg p.o., plasma concentration of RO5487624 was higher than its EC_90_ for a period of 7 and 10 hours, respectively. This estimation supplies a rationale of execution of an *in vivo* efficacy study of RO5487624.

### *In vivo* anti-influenza activities of RO5487624

**Figure 7:**
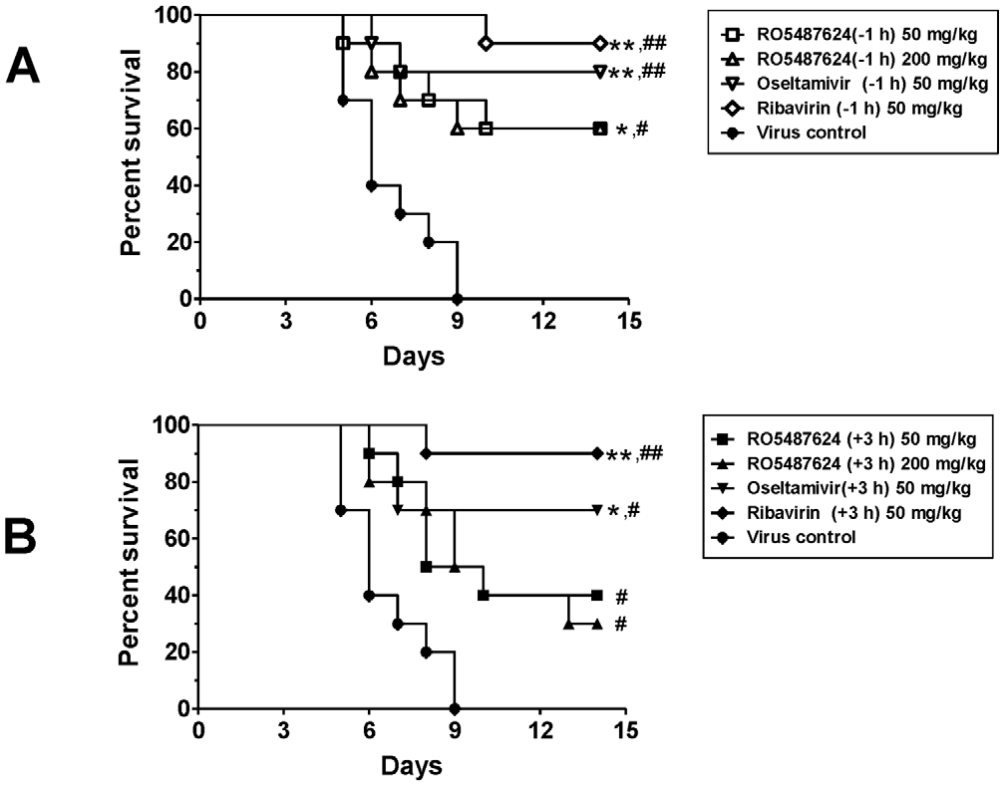
*In vivo* efficacy of RO5487624. 40 LD_50_ of A/FM/1/47 (H1N1) were used for viral challenge in all groups by intranasal inoculation. The first dosage of all treatments was given either 1 h before (A) or 3 h after (B) virus challenge (see details in Materials and Methods section). Mice in untreated control group were orally administrated with PBS twice a day for 7 days. Animals (n = 10 in each group) was monitored for 14 days starting from virus challenge. Data was analyzed with Chi-square for mortality rate, * *P*<0.05; ** *P*<0.001, and with Kaplan-Meier for mean survival day, # *P*<0.05; ## *P*<0.001, respectively.

With respect to *in vivo* efficacy of RO5487624, mice infected with 40 LD_50_ of an H1N1 strain were administrated with RO5487624 or other positive controls, ribavirin and oseltamivir, starting at either 1 h before or 3 h after virus challenge (see materials and methods). The efficacy of these compounds was evaluated on the basis of mean survival day and survival rate measured for 14 days. As shown in Fig. 7, RO5487624 displayed a significant efficacy in all treated groups in terms of mean survival day (*P*<0.05). Consistently, a previous *in vivo* experiment of RO5487624 with the same administration route and dosages as described in Fig. 7 showed a similar elongation of mean survival day (*P*<0.05), in which only post exposure prophylaxis (3 h after virus challenge, 17 LD_50_) of the compound was studied. In Fig. 7, prophylactic administration of RO5487624 and two positive controls displayed better efficacies than post exposure prophylaxis administration. RO5487624 significantly increased survival rate (*P*<0.05) when given 1 h prior to virus inoculation. These *in vivo* data suggest that RO5487624, as an HA fusion inhibitor, has a protective effect on mice that were lethally challenged with influenza H1N1 virus.

Before *in vivo* efficacy study, we administrated mice with RO5487624 orally at the dosages of 200 mg/kg and 50 mg/kg, respectively, twice a day for 7 days without virus challenge. Survival of the treated animals and body weight were monitored for 14 days starting from the first day of compound dosing. We did not find any animal death and statistically significant body weight changes in this experiment.

## Discussion

In this study, we showed that RO5464466 and RO5487624, two representative compounds of benzenesulfonamide, inhibit fusion as well as overall replication of H1N1 influenza viruses in both *in vitro* and *in vivo* models in which multiple rounds of virus infection and amplification occur.

Time course study of one cycle of influenza virus replication (Fig. 2) suggested that the benzenesulfonamides inhibited an early step of influenza replication but did not interfere with virus absorption or receptor binding step. The latter claim was supported by hemagglutination inhibition assay where the presence of the compound failed to show any inhibitory effect on virus-induced hemagglutination of chicken red blood cells. To further investigate the compound's mechanism of action, HA-mediated hemolysis assay of chicken red blood cells and trypsin sensitivity assay using purified HA were performed. As shown in Figure 3, RO5464466 completely blocked hemolysis triggered by a short exposure of virus-absorbed chicken red blood cells in low pH environments. Since virus absorption and HA conformational changes are two key events required for hemolysis, the blockage effect of the compound on hemolysis might be due to a halt of HA conformational changes. This hypothesis was confirmed in an HA trypsin sensitivity assay where characteristic changes of bromelain cleaved HA (BHA) were inhibited in the presence of RO5464466, rendering BHA resistant to trypsin digestion. In the literature, small molecules can bind to HA and block influenza fusion by two different mechanisms: stabilizing or destabilizing HA overall structures [5], [19]–[21], [24], [25], [30]. An HA destabilizer, represented by C22, inhibits virus fusion by prematurely triggering HA conformational changes [30]. Nevertheless, this effect cannot protect HA from trypsin digestion since HA conformational changes do occur in the presence of a destabilizer. Based on our data, RO5464466 and RO5487624 are HA stabilizers that abolish influenza virus fusion by blocking HA conformational changes in low pH environments.

To explore the therapeutic value of benzenesulfonamides, a plaque formation assay was employed where multiple rounds of influenza virus infection, replication, and death of infected cells were involved. RO5464466 can attenuate an established infection reflected by the results that no plaque or only small-sized plaques were observed when the compound was added 12 to 24 hours post infection. Furthermore, by inhibiting virus fusion, RO5464466 completely blocked the production of progeny influenza virus in an *in vitro* cell based system (Fig. 5, B) at a concentration of 3.16 µM, which is about 10 times the EC_50_ (CPE assay) of the compound. Consistent with its *in vitro* anti-influenza activities, a benzenesulfonamide compound exhibited an *in vivo* efficacy in mice that were challenged with an H1N1 virus strain. At this stage, *in vivo* efficacy of benzenesulfonamides represented by RO5487624 was weaker than that of ribavirin or oseltamivir. Besides differences of mechanism of action of these chemicals, limited exposure in plasma and lungs and high protein binding of RO5487624 might be reasons attributable to overall *in vivo* outcomes. Improvements on pharmacokinetic properties of benzenesulfonamides are required for its further development.

Recently, several studies have shown that neutralizing antibodies can be elicited against HA_2_, a more conserved part than HA_1_ of hemagglutinin [31]–[33]. These antibodies target the stem region of HA_2_ and can protect mice against challenge with influenza A viruses. Like HA small molecule inhibitors reported previously, antibody-mediated protection was also group-specific. For example, monoclonal antibody 12D1 elicited with an H3-based HA_2_ immunogen cannot prevent infections of group one influenza viruses [31]. In this report, benzenesulfonamides show activities to several influenza H1N1 strains. However, it is possible for this chemical series to behavior like other group specific HA inhibitors and block replication of other members of group one influenza viruses besides H1N1. Taken together, it might be necessary to combine two or more different types of small molecule inhibitors or antibodies against HA for a full coverage of the anti-influenza spectrum for potential clinical use.
